# Comparison of simultaneous integrated tumor bed boost and sequential boost during hypofractionated whole-breast irradiation after breast-conserving surgery

**DOI:** 10.1016/j.ctro.2025.100967

**Published:** 2025-04-25

**Authors:** Dan-Qiong Wang, Yu-Chun Song, Hao Jing, Hui Fang, Yong-Wen Song, Yue-Ping Liu, Jing Jin, Shu-Nan Qi, Yuan Tang, Ning-Ning Lu, Bo Chen, Ning Li, Yi-Rui Zhai, Wen-Wen Zhang, Xin Liu, Si-Ye Chen, Zhuan-Bo Yang, Guang-Yi Sun, Xu-Ran Zhao, Zi-Han Qiu, Ye-Xiong Li, Yu Tang, Shu-Lian Wang

**Affiliations:** a Department of Radiation Oncology, National Cancer Center/National Clinical Research Center for Cancer/Cancer Hospital, Chinese Academy of Medical Sciences and Peking Union Medical College, Beijing 100021, China; b Department of Radiation Oncology, Cancer Hospital & Shenzhen Hospital, Chinese Academy of Medical Sciences and Peking Union Medical College, Shenzhen, China

**Keywords:** Breast cancer, Simultaneous integrated tumor bed boost, Sequential tumor bed boost, Hypofractionated whole-breast irradiation

## Abstract

**Background and purpose:**

This study aimed to compare the safety and efficacy of simultaneous integrated boost (SIB) and sequential boost (SeB) during hypofractionated WBI.

**Materials and methods:**

This study analyzed data from two prospective studies, including 1,132 patients with pT1-3 N0-3 M0 breast cancer, of whom 775 received SIB and 357 received SeB. The prescribed dose was 43.5 Gy in 15 fractions to whole breast and/or nodal region, with either 49.5 Gy in 15 fractions (SIB) or 8.7 Gy in 3 fractions (SeB) delivered to tumor bed. Outcomes analyzed included survival outcomes, treatment-related toxicities, and cosmetic outcomes.

**Results:**

The 5-year outcomes were local control rates of 97.8 % vs. 98.8 % (*p* = 0.12), locoregional control rates of 97.7 % vs. 97.1 % (*p* = 0.72), disease-free survival of 94.1 % vs. 93.1 % (*p* = 0.71), overall survival of 97.4 % vs. 97.1 % (*p* = 0.88), and breast-specific survival of 98.2 % vs. 97.5 % (*p* = 0.43) for SIB versus SeB, respectively. After stabilized inverse probability of treatment weighting, differences between groups remained non-significant. Rates of fair or poor cosmetic outcomes before and after radiotherapy were lower in the SIB group, but there was no difference in cosmetic deterioration (9.8 % vs. 7.6 %, *p* = 0.22). Grade 2 or higher toxicities, including skin toxicity, pneumonitis, breast swelling, pain, induration, lymphedema, and shoulder mobility issues, were comparable between groups.

**Conclusion:**

SIB is a viable alternative to SeB, offering comparable toxicity profiles and survival outcomes while shortening treatment duration. Longer follow-up is warranted to assess long-term outcomes.

## 1 Background

For patients with early invasive breast cancer, breast-conserving surgery combined with adjuvant radiotherapy has replaced total mastectomy as the preferred treatment when appropriate. According to the *meta*-analysis conducted by the Early Breast Cancer Trialists’ Collaborative Group, adjuvant radiotherapy following breast-conserving surgery reduces breast cancer mortality by one-sixth, decreases the 10-year first recurrence rate (locoregional or distant metastasis) from 35.0 % to 19.3 %, and lowers the 15-year breast cancer mortality rate from 25.2 % to 21.4 % [1].

As most ipsilateral breast tumor recurrences occur in the primary tumor bed, sequential boost (SeB) in conventionally fractionated whole-breast irradiation (CF-WBI) has been shown to significantly reduce 20-year ipsilateral breast tumor recurrence rates and the need for salvage total mastectomy compared to no-boost treatment (16.4 % vs. 12.0 %), establishing SeB as an integral component of WBI [2].

Hypofractionated WBI (HF-WBI) has demonstrated efficacy comparable to CF-WBI, without an increase in toxicity [[3], [4], [5]]. Furthermore, the START B study revealed that the incidence of breast shrinkage, telangiectasia, and breast swelling was significantly lower in the HF-WBI group compared to CF-WBI [4]. Although HF-WBI shortens the overall treatment duration, the inclusion of conventionally fractionated SeB (10 Gy/5 fractions) undermines this time-efficient advantage. Studies by Wang et al. [6] and Shaitelman et al. [7] have reported that hypofractionated SeB (8.7–10 Gy/3–4 fractions) with HF-WBI achieved local control (LC) and cosmetic outcomes comparable to those of conventionally fractionated SeB with CF-WBI.

The simultaneous integrated boost (SIB) technique delivered the tumor boost concurrently with WBI by increasing the dose per fraction. This approach presents an appealing alternative to SeB, avoiding unnecessary prolongation of treatment. With the widespread use of tumor bed metal clips and intensity-modulated radiotherapy (IMRT), precise localization of the tumor bed and improved dose homogeneity have provided a strong foundation for the adoption of SIB. However, the efficacy and safety of SIB compared to SeB, particularly in the context of HF-WBI, remain uncertain.

The presented study compared survival outcomes, toxicities, and breast cosmetic outcomes between the SIB and SeB groups in early invasive breast cancer patients treated with HF-WBI.

## 2 Materials and methods

### 2.1 Patients

This is a single-center study, including 357 patients receiving HF-WBI with SeB between 2010 and 2015 as part of a phase III randomized study (NCT01413269) [6] and 775 patients receiving HF-WBI with SIB in a phase II prospective study (NCT03320421) between 2016 and 2018. Patients were eligible for inclusion if they had pT1-3 N0-3 M0 breast cancer and had undergone lumpectomy with axillary dissection or sentinel node biopsy with negative margins. Both studies received approval from the institutional review board of our hospital (approval numbers CH-BC-013 and NCC2016YQ-19), and informed consent was obtained from all participants.

### 2.1 Radiotherapy

All patients received HF-WBI ± regional nodal irradiation (RNI) at a dose of 43.5 Gy in 15 fractions. The tumor bed was simultaneously treated with 49.5 Gy in 15 fractions in SIB group and sequentially with 8.7 Gy in 3 fractions in SeB group. Assuming a tumor α/β ratio of 4, the equivalent dose in 2 Gy fractions (EQD2) was 50.0 Gy for HF-WBI and RNI, while the doses for SIB and SeB reached 60.2 Gy and 60.0 Gy, respectively.

The clinical target volume (CTV) for WBI included the entire breast and the fascia of the pectoralis major muscle. RNI CTV encompassed the supraclavicular, infraclavicular, and level II axillary nodal regions. The planning target volume (PTV) was derived by expanding the CTV by 0.5 cm for RNI and by 0.5–1.0 cm for WBI. The tumor bed, identified based on seroma, surgical changes, and metal clips, was expanded by 1 cm for PTV boost in the SeB group. For the SIB group, the CTV boost was expanded from the tumor bed by 1 cm without exceeding the WBI CTV, and an additional 0.5 cm margin was added to define the PTV boost for patient setup uncertainties. All PTVs were restricted to a depth of 0.5 cm beneath the skin surface.

Radiotherapy was administered using computed tomography-based three-dimensional conformal radiotherapy (3DCRT), tangential IMRT, or volumetric modulated arc therapy (VMAT). The prescribed dose covered 95 % of the PTV for all groups except SeB, which was delivered with electrons, with the prescribed dose covering 90 % of the PTV boost. In both studies, organs at risk, including the heart, left anterior descending coronary artery, ipsilateral lung, contralateral breast, and spinal cord PRV, were contoured. Dose constraints followed institutional protocols: heart (Dmean ≤ 4 Gy, V26 < 20 %, V35 < 10 %); left anterior descending coronary artery (Dmean < 25 Gy); ipsilateral lung (Dmean ≤ 13 Gy, V17 < 25 %); contralateral breast (Dmean < 5 Gy, V20 < 5 %); spinal cord PRV (Dmax < 32 Gy). Cone-beam computed tomography or electronic portal imaging device was employed to verify setup accuracy before treatment for the first 3–5 sessions and weekly thereafter.

### 2.3 Outcomes

The outcomes assessed included LC, locoregional control (LRC), disease-free survival (DFS), overall survival (OS), breast cancer-specific survival (BCSS), treatment-related toxicities, and breast cosmetic outcomes. LC was defined as the time until recurrence in the ipsilateral breast or the last follow-up if no event occurred. LRC was defined as the time until recurrence in the ipsilateral breast, axillary, supraclavicular, infraclavicular, or internal mammary lymph nodes, or the last follow-up if no event occurred. DFS included local recurrence, locoregional recurrence, distant metastasis, or death from any cause, whichever occurred first. OS and BCSS were defined as death from any cause and death from breast cancer, respectively.

Treatment-related toxicities were assessed during treatment and follow-up visits using the Common Toxicity Criteria for Adverse Events version 3.0. Patients were followed up every 3 months for 2 years after radiotherapy, then every 6 months from 3 to 5 years, and yearly thereafter. Breast cosmetic outcomes were evaluated using patient self-reported assessments based on the Harvard 4-point scale. Cosmetic outcomes before radiotherapy, 5 years after radiotherapy, and the extent of deterioration post-radiotherapy were analyzed. Since late toxicities are time-dependent, all toxicity analyses were capped at 5 years of follow-up.

### 2.4 Statistical analysis

Patient characteristics were summarized as frequencies and percentages. Chi-square or Fisher’s exact tests were used to compare baseline characteristics and the incidence of adverse outcomes, including fair or poor cosmetic results, breast induration, grade ≥ 2 toxicities between groups. Bonferroni correction is used for multiple group comparisons. To address baseline imbalances, stabilized inverse probability of treatment weighting (IPTW) was applied to construct a matched cohort for analysis. IPTW variables were selected based on significant baseline differences or clinical relevance to prognosis. Adjusted covariates included age, BMI, tumor location, surgical approach, histologic type, ductal carcinoma *in situ* component, TNM stage, histologic grade, lymphovascular invasion, molecular subtype, chemotherapy, HER2 status and anti-HER2 targeted therapy, and hormone receptor status and endocrine therapy.

The Kaplan-Meier method was used to estimate LC, LRC, DFS, OS, and BCSS rates, with statistical comparisons made using the log-rank test. Statistical significance was set at *p* < 0.05. Analyses were performed using SPSS Statistics v 26.0 (IBM Corp., Armonk, NY, USA) and R software v4.1.2 (http://www.r-project.org/).

## 3 Results

### 3.1 Characteristics

The clinical and pathological characteristics of the entire cohort, along with comparisons between patients receiving SIB and SeB, are summarized in Table 1. Compared with the SeB group, patients in the SIB group were significantly more likely to present with T2–3 stage, N1–3 stage, ductal carcinoma *in situ* component, lymphovascular invasion, and to have undergone neoadjuvant or adjuvant chemotherapy, anti-HER2 targeted therapy, and endocrine therapy. Among those receiving chemotherapy, 64.9 % of the SIB group and 74.8 % of the SeB group received taxane-containing regimens, with a median of six cycles in both groups. Of the 928 patients with positive estrogen receptor and/or progesterone receptor, 884 (95.3 %) underwent endocrine therapy, while 157 of the 194 HER2-positive patients (80.9 %) received anti-HER2 targeted therapy.

**Table 1:** Clinical, tumor, and treatment characteristics of the entire cohort and the two groups.

**Characteristic**	**Entire cohort**	**SeB**	**SIB**	*P*-value*
	**N (%)**	**N (%)**	**N (%)**	
Number of patients	**1132**	**357**	**775**	
Age (y)				0.724
≤ 40	314 (27.7)	102 (28.6)	212 (27.4)	
> 40	818 (72.3)	255 (71.4)	563 (72.6)	
BMI (kg/m^2^)				0.013
< 18.5	38 (3.4)	13 (3.6)	25 (3.2)	
18.5–24.9	678 (59.9)	235 (65.8)	443 (57.2)	
≥ 25	416 (36.7)	109 (30.5)	307 (39.6)	
Tobacco consumption				0.111
No	1121 (99.0)	351 (98.3)	770 (99.4)	
Yes	11 (1.0)	6 (1.7)	5 (0.6)	
Diabetes				0.014
No	1055 (93.2)	323 (90.5)	732 (94.5)	
Yes	77 (6.8)	34 (9.5)	43 (5.5)	
Hypertension				0.150
No	955 (84.4)	293 (82.1)	662 (85.4)	
Yes	177 (15.6)	64 (17.9)	113 (14.6)	
Tumor laterality				0.405
Left	585 (51.7)	191 (53.5)	394 (50.8)	
Right	547 (48.3)	166 (46.5)	381 (49.2)	
Tumor location				0.305
Medial or central	457 (40.4)	152 (42.6)	305 (39.4)	
Other quadrants	675 (59.6)	205 (57.4)	470 (60.6)	
Surgery				0.052
Lumpectomy and axillary dissection	400 (35.5)	143 (40.1)	259 (33.4)	
Lumpectomy and sentinel node biopsy	730 (64.5)	214 (59.9)	516 (66.6)	
Tumor bed metal clips				< 0.001
No	124 (11.0)	60 (16.8)	64 (8.3)	
Yes	1008 (89.0)	297 (83.2)	711 (91.7)	
Histologic type				0.002
Ductal invasive carcinoma	980 (86.6)	320 (89.6)	660 (85.2)	
Lobular invasive carcinoma	53 (4.7)	5 (1.4)	48 (6.2)	
Others	99 (8.7)	32 (9.0)	67 (8.6)	
Ductal carcinoma *in situ* component				< 0.001
No	721 (63.7)	266 (74.5)	455 (58.7)	
Yes	411 (36.3)	91 (25.5)	320 (41.3)	
T stage†				< 0.001
T1	827 (73.1)	287 (80.4)	540 (69.7)	
T2	299 (26.4)	70 (19.6)	229 (29.5)	
T3	6 (0.5)	0 (0)	6 (0.8)	
N stage†				0.002
N0	833 (73.6)	285 (79.8)	548 (70.7)	
N1	225 (19.9)	60 (16.8)	165 (21.3)	
N2	56 (4.9)	11 (3.1)	45 (5.8)	
N3	18 (1.6)	1 (0.3)	17 (2.2)	
Stage group (AJCC 7.0) †				0.006
IA	650 (57.4)	239 (66.9)	411 (53.0)	
IIA	324 (28.6)	87 (24.4)	237 (30.6)	
IIB	81 (7.2)	19 (5.3)	62 (8.0)	
III	77 (6.8)	12 (3.4)	65 (8.4)	
Histologic grade				0.945
I	94 (8.3)	30 (8.4)	64 (8.3)	
II	596 (52.7)	191 (53.5)	405 (52.3)	
III	319 (28.2)	100 (28.0)	219 (28.3)	
Unknown	123 (10.9)	36 (10.1)	87 (11.2)	
Lymphovascular invasion				< 0.001
No	921 (81.4)	320 (89.6)	601 (77.5)	
Yes	211 (18.6)	37 (10.4)	174 (22.5)	
Extracapsular extension				0.172
No	1106 (97.7)	352 (98.6)	754 (97.3)	
Yes	26 (2.3)	5 (1.4)	21 (2.7)	
Molecular subtype				0.115
HR + HER2−	780 (68.9)	258 (72.3)	522 (67.4)	
HER2+	194 (17.1)	60 (16.8)	134 (17.3)	
Triple-negative	158 (14.0)	39 (10.9)	119 (15.4)	
Chemotherapy				< 0.001
None	297 (26.2)	111 (31.1)	186 (24.0)	
Neoadjuvant chemotherapy	25 (2.2)	0 (0)	25 (3.2)	
Neoadjuvant and adjuvant chemotherapy	8 (0.7)	1 (0.3)	7 (0.9)	
Adjuvant chemotherapy	802 (70.8)	245 (68.6)	558 (72.0)	
Anti-HER2 targeted therapy‡				< 0.001
No	37 (19.1)	27 (45.0)	10 (7.5)	
Yes	157 (80.9)	33 (55.0)	124 (92.5)	
Endocrine therapy§				0.007
No	44 (4.7)	9 (2.9)	35 (5.6)	
Yes	884 (95.3)	299 (97.1)	585 (94.4)	

Abbreviations: AJCC = American Joint Committee on Cancer; BMI = body mass index; HER2 = human epidermal growth factor receptor 2; HR = hormone receptor; SeB = sequential boost; SIB = simultaneous integrated boost.

* Comparison between SeB and SIB groups.

† For patients who received neoadjuvant chemotherapy, we used whichever stage was higher (clinical or pathological) to reflect the actual tumor burden.

‡ For HER2-positive patients.

§ For hormone receptor positive patients.

RNI was performed in 123 (15.9 %) patients in the SIB group compared with 11 (3.1 %) in the SeB group. In the SIB group, RNI included the supraclavicular/infraclavicular region in 102 (13.2 %) patients, the supraclavicular/infraclavicular region plus the axilla in 14 (1.8 %) patients, the supraclavicular/infraclavicular region plus the internal mammary region in five (0.6 %) patients, and one patient (0.1 %) receiving all three. Additionally, one patient (0.1 %) had RNI to the axilla alone. In contrast, RNI in the SeB group included the supraclavicular/infraclavicular region in 11 (3.1 %) patients. The median PTV boost was 105.1 mL (range: 22.0–492.3 mL) in the SIB group and 115.5 mL (range: 29.8–781.2 mL) in the SeB group, with no significant difference between the two groups (p = 0.236). WBI and RNI were predominantly performed using IMRT or VMAT in 1,123 (99.8 %) patients, with 3DCRT used in only nine (0.8 %) patients. For tumor bed boosts, IMRT or VMAT was used in all 775 SIB patients. In the SeB group, electrons were used in 336 (94.1 %) patients, while IMRT or VMAT was used in the remaining 21 (5.9 %).

### 3.2 Survival outcomes

**Fig. 1:**
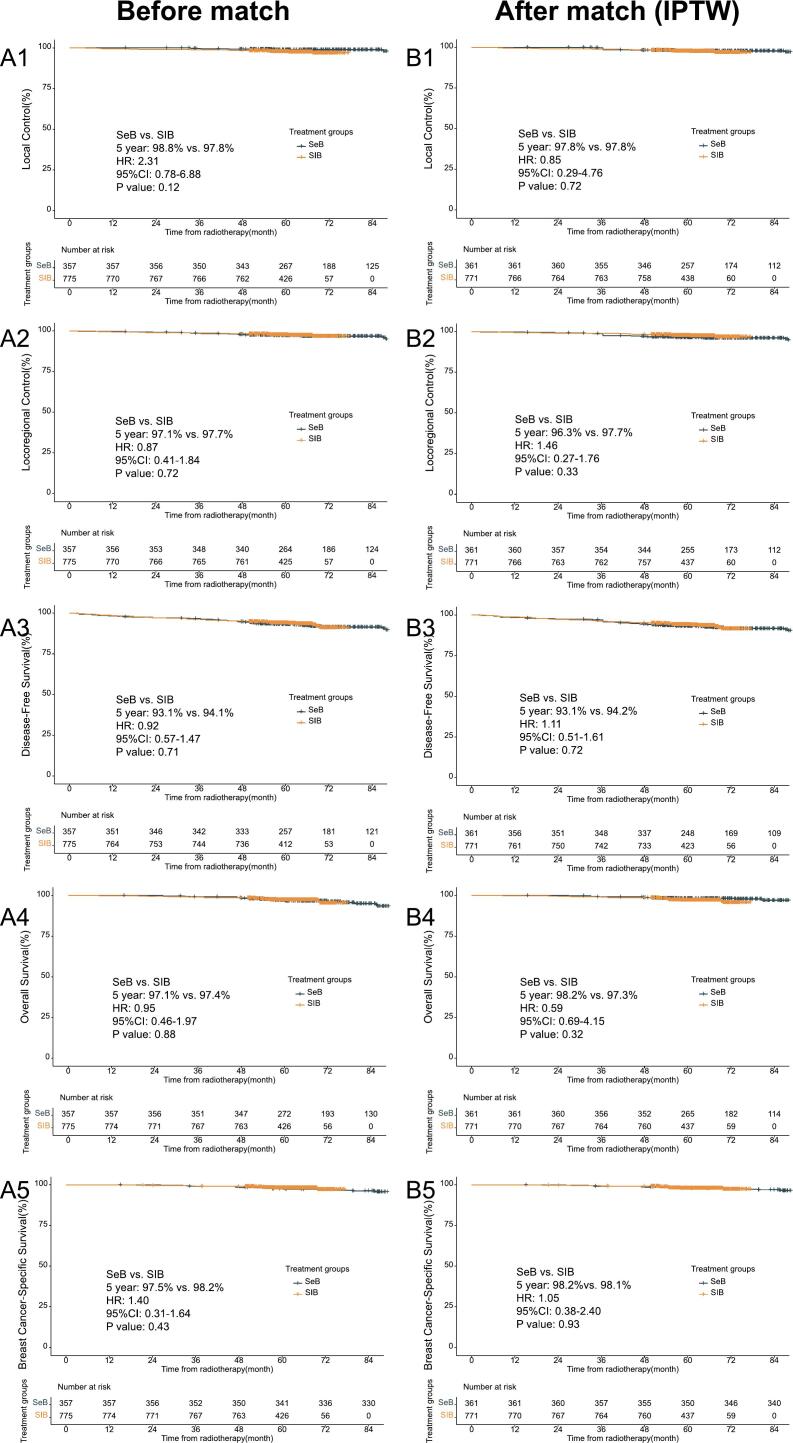
Comparison of local control (LC), locoregional control (LRC), disease-free survival (DFS), overall survival (OS), and breast-specific survival (BSS) between patients receiving sequential boost (SeB) and those receiving simultaneous integrated boost (SIB). Panels A1–A5 represent LC, LRC, DFS, OS, and BSS outcomes before matching, respectively. Panels B1–B5 represent LC, LRC, DFS, OS, and BSS outcomes after inverse probability of treatment weighting (IPTW), respectively.

The median follow-up period was 65 months (range: 15–146 months) for the entire cohort, 61 months (range: 47–77 months) for the SIB group, and 107 months (range: 15–146 months) for the SeB group. For the entire cohort, the 5-year rates for LC, LRC, DFS, OS, and BCSS were 98.1 %, 97.5 %, 93.8 %, 97.3 %, and 97.9 %, respectively. No significant differences were observed between the SIB and SeB groups in 5-year LC (97.8 % vs. 98.8 %, *p* = 0.12), LRC (97.7 % vs. 97.1 %, *p* = 0.72), DFS (94.1 % vs. 93.1 %, *p* = 0.71), OS (97.4 % vs. 97.1 %, *p* = 0.88), or BCSS (98.2 % vs. 97.5 %, *p* = 0.43) (Fig. 1). After stabilized IPTW, the weighted patient numbers in the SIB and SeB groups were 770.8 and 361, respectively (Supplementary Table 1, Supplementary Fig. 1). Due to the nature of IPTW adjustment, the weighted sample size may not be an integer, as some patients contribute partial weights to the final analysis. No significant differences were found between the SIB and SeB groups for 5-year LC (97.8 % vs. 97.8 %, *p* = 0.72), LRC (97.7 % vs. 96.3 %, *p* = 0.33), DFS (94.2 % vs. 93.1 %, *p* = 0.72), OS (97.3 % vs. 98.2 %, *p* = 0.32), or BCSS (98.1 % vs. 98.2 %, *p* = 0.93).

The patterns of recurrence are summarized in Table 2. Among the 37 patients with locoregional recurrence, 16 (43.3 %) experienced ipsilateral breast recurrence (IBR) with regional lymph node recurrence, 12 (32.4 %) had isolated IBR, and 9 (24.3 %) had regional lymph node recurrence. A total of 42 patients died during the follow-up period, of whom 32 (76.2 %) died of breast cancer, nine (21.4 %) of other diseases, and one (2.4 %) of a second malignancy. Of the 53 patients diagnosed with second malignancies, 14 were in the thyroid, 11 in the contralateral breast, 11 in the lung, eight in the digestive system, four in the kidney, three in the reproductive system, and two in the brain.

**Table 2:** The patterns of recurrence in the entire cohort, SeB, and SIB groups.

**The patterns of recurrence**	**Number (%)**		
	**Entire group**	**SeB**	**SIB**
Local recurrence (ipsilateral breast recurrence)	28	10	18
Non-index quadrant	14 (50.0)	5 (50.0)	9 (50.0)
Index quadrant	13 (46.4)	5 (50.0)	8 (44.4)
Both the index and non-index quadrants	1 (3.6)	0 (0)	1 (5.6)
Regional lymph node recurrence	25	16	15
Supraclavicular/infraclavicular region	12 (48.0)	6 (37.5)	6 (40.0)
Ipsilateral axillary region	10 (40.0)	4 (25.0)	6 (40.0)
Internal mammary lymph nodes	9 (36.0)	6 (37.5)	3 (20.0)
Distant metastasis	69	37	32
Isolated distant metastases	49 (71.0)	26 (70.3)	23 (71.9)
Distant metastases with concurrent locoregional recurrence	20 (29.0)	11 (29.7)	9 (28.1)

Abbreviations: SeB = sequential boost; SIB = simultaneous integrated boost.

### 3.3 Toxicity and cosmetic outcomes

Table 3 summarizes treatment-related toxicities, most of which were grade 1 or 2. Three patients experienced grade 3 ischemic heart disease or acute myocardial infarction: two had left-sided tumors, and one had a right-sided tumor. All patients experienced precordial pain, were diagnosed with vascular stenosis via coronary angiography, and recovered well after surgery. In the SeB group, two patients developed grade 3 skin toxicity. Bonferroni correction indicated higher grade 1 pneumonitis and breast pain but lower grade 1 skin toxicity in the SIB group compared to the SEB group. While, since grade 1 toxicities were mild and did not require clinical intervention, we focused our comparison on clinically relevant grade 2 or higher toxicities (Table 4). There was no significant difference observed between the SIB and SeB groups in grade 2 or higher skin toxicity, pneumonitis, breast swelling, breast pain, lymphedema, shoulder mobility issues, or ischemic heart disease. The SIB group did not show a higher incidence of breast induration, either in the scarred or non-scarred areas, compared with the SeB group.

**Table 3:** Toxicities and breast cosmetic outcomes in the entire cohort, SeB, and SIB groups.

**Adverse events, grade**	**Number (%)**			*P*-value*
	**Entire cohort**	**SeB**	**SIB**	
Skin toxicity				< 0.001
0	303 (26.8)	35 (9.8)	268 (34.6)	
1	779 (68.8)	311 (87.1)	468 (60.4)	
2	48 (4.2)	9 (2.5)	39 (5.0)	
3	2 (0.2)	2 (0.6)	0 (0)	
Pneumonitis/pulmonary infiltrates				< 0.001
0	744 (65.7)	314 (88.0)	430 (55.5)	
1	367 (32.4)	37 (10.4)	330 (42.6)	
2	21 (1.9)	6 (1.7)	15 (1.9)	
Breast swelling				0.123
0	1030 (91.0)	333 (93.3)	697 (89.9)	
1	77 (6.8)	20 (5.6)	57 (7.4)	
2	25 (2.2)	4 (1.1)	21 (2.7)	
Breast pain				< 0.001
0	991 (87.5)	337 (94.4)	654 (84.4)	
1	129 (11.4)	19 (5.3)	110 (14.2)	
2	12 (1.1)	1 (0.3)	11 (1.4)	
Lymphedema				0.525
0	1016 (89.8)	323 (90.5)	693 (89.4)	
1	104 (9.2)	32 (9.0)	72 (9.3)	
2	12 (1.0)	2 (0.6)	10 (1.3)	
Shoulder mobility issues				0.942
0	1106 (97.7)	348 (97.5)	758 (97.8)	
1	23 (2.0)	8 (2.2)	15 (1.9)	
2	3 (0.3)	1 (0.3)	2 (0.3)	
Brachial plexus neuropathy				0.128
0	1127 (99.6)	357 (100)	770 (99.4)	
1	5 (0.4)	0 (0)	5 (0.6)	
2	0 (0)	0 (0)	0 (0)	
Ischemic heart disease				0.510
0	1120 (98.9)	352 (98.6)	768 (99.2)	
1	8 (0.7)	3 (0.8)	5 (0.6)	
2	1 (0.1)	0 (0)	1 (0.1)	
3	3 (0.3)	2 (0.6)	1 (0.1)	
Pericarditis				0.924
0	1132 (100.0)	357 (100.0)	775 (100.0)	
1	0 (0)	0 (0)	0 (0)	
2	0 (0)	0 (0)	0 (0)	
Rib fracture				
0	1030 (91.0)	355 (99.4)	771 (99.5)	
1	6 (0.5)	2 (0.6)	4 (0.5)	
2	0 (0)	0 (0)	0 (0)	
Breast induration				0.558
No	975 (86.1)	313 (87.7)	662 (85.4)	
Scar area	118 (10.4)	30 (8.4)	88 (11.4)	
Outside the scarred area	39 (3.4)	14 (3.9)	25 (3.2)	
Global cosmetic outcome				< 0.001
Excellent	59 (5.2)	47 (13.2)	12 (1.5)	
Good	1017 (89.8)	273 (76.5)	744 (96.0)	
Fair	51 (4.5)	32 (9.0)	19 (2.5)	
Poor	5 (0.4)	5 (1.4)	0 (0)	

Abbreviations: SeB = sequential boost; SIB = simultaneous integrated boost.

* Comparison between SeB and SIB groups.

**Table 4:** Grade 2 or higher toxicities and breast cosmetic outcomes in the entire cohort, SeB, and SIB groups.

**Adverse events**	**Number (%)**			*P*-value*
	**Entire cohort**	**SeB**	**SIB**	
Skin toxicity	50 (4.4)	11 (3.1)	39 (5.0)	0.138
Pneumonitis/pulmonary infiltrates	21 (1.9)	6 (1.7)	15 (1.9)	0.768
Breast swelling	25 (2.2)	4 (1.1)	21 (2.7)	0.091
Breast pain	12 (1.1)	1 (0.3)	11 (1.4)	0.117
Lymphedema	12 (1.1)	2 (0.6)	10 (1.3)	0.359
Shoulder mobility issues	3 (0.3)	1 (0.3)	2 (0.3)	1.000
Ischemic heart disease	4 (0.4)	2 (0.6)	2 (0.2)	0.595
Breast induration				
Inside and outside the scar area	157 (13.9)	44 (12.3)	113 (14.6)	0.308
Fair or poor cosmetic outcome				
Before radiotherapy	50 (4.4)	42 (11.8)	26 (3.4)	< 0.001
After radiotherapy	56 (4.9)	37 (10.4)	37 (4.8)	< 0.001
Cosmetic outcome deterioration	103 (9.1)	27 (7.6)	76 (9.8)	0.223

Abbreviations: SeB = sequential boost; SIB = simultaneous integrated boost.

* Comparison between SeB and SIB groups.

Cosmetic outcomes were significantly better in the SIB group than the SeB group, with a lower incidence of fair or poor breast cosmesis observed before and after radiotherapy. However, the rate of cosmesis deterioration did not differ significantly between the groups. Breast cosmesis was unchanged or improved in 699 (90.2 %) patients in the SIB group and 330 (92.4 %) in the SeB group, while it deteriorated in 76 (9.8 %) and 27 (7.6 %) patients, respectively. Among those with deteriorated cosmesis following radiotherapy, two patients in the SeB group transitioned from excellent or good to poor. Additionally, 73 (9.4 %) patients in the SIB group and 24 (6.7 %) in the SeB group transitioned from excellent or good to fair, while three (0.4 %) patients in the SIB group and one (0.3 %) in the SeB group transitioned from excellent to good.

## 4 Discussion

This study prospectively evaluated the efficacy and safety of SIB and SeB during HF-WBI in breast cancer patients following breast-conserving surgery. Both the SIB and SeB groups demonstrated low relapse rates and favorable 5-year survival outcomes. The prevalence of grade ≥ 2 treatment-related toxicities was minimal, with no significant differences between the two groups. Additionally, there was no difference in the rate of breast cosmesis deterioration between the groups, suggesting that SIB does not adversely affect cosmetic outcomes. Therefore, SIB may serve as an effective alternative to SeB, offering comparable efficacy and safety.

The primary advantage of SIB during HF-WBI lies in its higher per-fraction dose and shorter overall treatment duration. Given the breast cancer α/β ratio of approximately 3–4 Gy [3], which reflects sensitivity to fraction size, hypofractionation is biologically reasonable in the SIB setting. Compared to SeB, SIB reduces the length and cost of radiotherapy, alleviating patients’ time and financial burdens while improving compliance with adjuvant treatment. Additionally, SIB offers improved dose homogeneity across the entire breast. Van Der Laan et al. [8] demonstrated that SIB reduced the PTV volume receiving ≥ 107 % of the prescription dose by 20 % and the volume within the PTV and outside the PTV boost receiving ≥ 95 % of the prescription dose by 54 %. Advances in radiotherapy technologies, such as multi-beam IMRT and VMAT, have further enhanced dose homogeneity within the target region and improved treatment volume coverage while minimizing exposure to organs at risk [[9], [10], [11], [12]].

There were no differences in 5-year survival outcomes between the SIB and SeB groups in this study and these results remained consistent after applying IPTW. Limited literature exists on head-to-head survival comparisons between SIB and SeB during HF-WBI. Appendix A, [35], [36], [37], [38], [39], [40], [41], [42], [43], [44], [45], [46], [47], [48] listed the related studies evaluating SIB during HF-WBI, reporting favorable 5-year LRC (95.3–99 %) and acceptable toxicity profiles with dose regimens ranging from 40–43.2 Gy in 15–16 fractions to WBI and 44–52.8 Gy in 15–16 fractions to SIB, including three phase III studies: the IMPORT HIGH trial [13], the NRG RTOG 1005 trial [14] and the HYPOSIB-trial [15].The IMPORT HIGH trial [13] showed the test and control groups all achieved high LC rates (96.8–98.1 %), however, the 53 Gy SIB offered no LC advantage and was associated with increased moderate or marked breast induration (15.5 % vs. 11.5 %, *p* = 0.02), whereas the 48 Gy SIB demonstrated non-inferiority in LC and similar breast induration rate compared to the control. This emphasized precise biologically effective dose estimation in hypofractionated radiotherapy, as illustrated by the normalized dose–response gradient. With an estimated γ50 of 1.8, a 5 % dose escalation is projected to increase the proportion of patients with significant changes in breast appearance by 9 % [16].

The present study, along with previous investigations, suggests that SIB does not increase grade 2 or higher toxicity. Additionally, some studies [[17], [18], [19], [20]] even consistently showed significantly lower rates of grade ≥ 2 skin toxicity in HF-WBI with SIB compared to CF-WBI with SeB. Similarly, in a comparative study of SIB and SeB during HF-WBI, Paelinck et al. [21] reported significantly higher rates of grade ≥ 2 skin toxicity in the SeB arm (45.8 % vs. 28.9 %). The reduction in acute toxicity observed with SIB may be attributed to improved dose distribution, a shorter radiotherapy course, and a lower total dose, particularly as early-responding tissues with higher α/β ratios are more sensitive to total dose. Further, dosimetric constraints have been identified as key factors influencing toxicity outcomes. De Rose et al. [22] demonstrated that receiving at least 20 Gy of treated skin exceeding 400 cm^3^ and a boost volume receiving 105 % of the prescription dose exceeding 5 cm^3^ were independent predictors of grade ≥ 2 skin toxicity in patients undergoing SIB during HF-WBI. In present study, incidence of grade 1 pneumonitis was higher in the SIB group, likely due to more frequent CT-based follow-ups, while the SeB group, enrolled earlier, primarily underwent chest X-rays. The higher incidence of grade 1 pain may be related to the increased scatter dose to normal breast tissue from intensity-modulated techniques compared to electron boost [23]. Future studies will further explore the dose–effect relationship of radiotherapy-related toxicities and clarify the dose parameter differences between groups.

Breast induration, another critical endpoint, is associated with tumor bed boost in a dose-dependent manner, as shown in the EORTC 22881–10882 trial [24]. However, the incidence of induration was not significantly different between SIB and SeB [13,16,19,25]. The review also indicates that the probability of grade ≥ 2 breast induration in SIB patients is relatively low, ranging from 1 % to 9 % [26]. A predictive model for grade ≥ 2 breast induration in patients undergoing CF-WBI with SIB identified patient age, breast volume receiving at least 55 Gy, and the maximum dose to the breast as significant factors [27]. This highlights the importance of dose homogeneity in mitigating adverse outcomes.

Cosmetic outcomes were significantly better in the SIB group in the current study compared to the SeB group. Further analysis revealed that these differences predated radiotherapy, potentially reflecting advances in surgical techniques over time. Cosmesis deterioration suggested that the boost type did not affect cosmetic outcomes, consistent with previous reports [15,20,[28], [29], [30]]. The cosmetic evaluation in this study relied on patient self-reports using the Harvard scale, whereas trials such as IMPORT HIGH employed photographic assessment, scoring on a 3-point ordinal scale. In IMPORT HIGH, the incidence of mild or marked changes in breast appearance at 5 years ranged from 24.4 % to 36.8 %, without significant differences between arms.

Factors influencing poor cosmesis include older age [31]. larger tumor volume [31,32], poorer pre-radiotherapy cosmesis [33], adjuvant chemotherapy [33], large breast volume [22], large boost volume [22,32], higher boost doses [33], dose inhomogeneity [31], and photon boost [33]. Accurate tumor bed localization using metal clips and advanced technology to enhance dose uniformity may improve cosmetic outcomes. De Rose et al. [22] reported that a breast volume ≥ 1000 cm^3^ impacted 2-year cosmesis, while a boost volume > 70 cm^3^ influenced 5-year outcomes, suggesting that HF-WBI with SIB may be feasible for patients with larger breasts, pending further long-term validation.

This study had several limitations. First, the non-randomized design resulted in the SIB group having more risk factors; thus, IPTW was used to balance baseline characteristics. Second, the SIB group had a higher proportion of patients’ tumor bed treated with IMRT/VMAT, while the SeB group with electrons. Patel et al. [34] analyzed the factors associated with skin toxicity in patients with large breasts undergoing HF-WBI, where 31.2 % of patients received photon boosts and 68.8 % received electron boosts. Multivariate analysis showed no association between photon or electron boosts and grade ≥ 2 skin toxicity. Third, the SeB group was enrolled earlier than the SIB group, limiting toxicity analyses to a 5-year follow-up. As the follow-up period was relatively short, necessitating longer-term evaluations to confirm cosmetic outcomes. Nonetheless, this study is one of the few prospective investigations comparing SIB and SeB during HF-WBI with a large sample size.

## 5 Conclusions

IMRT/VMAT SIB and electron SeB had similar efficacy and grade 2 or higher toxicity in patients undergoing HF-WBI after breast-conserving surgery. SIB was well tolerated overall, despite the higher incidence of grade 1 breast pain. Cosmesis impairment after radiotherapy was a rare event and it was comparable between the two groups. SIB offers a shortened treatment duration, which may enhance patient compliance and quality of life. The findings from this study support the feasibility of SIB as an alternative to SeB, although longer follow-up is warranted to validate these results.

## Statistician

Dan-Qiong Wang, Department of Radiation Oncology, National Cancer Center/National Clinical Research Center for Cancer/Cancer Hospital, Chinese Academy of Medical Sciences and Peking Union Medical College, 17 Panjiayuan nanli, Chaoyang District, Beijing 100021, China.

## Funding

Chinese Academy of Medical Sciences Innovation Fund for Medical Sciences [2021-I2M-1–014] and Noncommunicable Chronic Diseases-National Science and Technology Major Project [2023ZD0502200].

## Declaration of Competing Interest

The authors declare that they have no known competing financial interests or personal relationships that could have appeared to influence the work reported in this paper.

## Data Availability

The data used in the study analyses can be made available by the corresponding author on reasonable request.
