# Supplementary material for: Comparison of simultaneous integrated tumor bed boost and sequential boost during hypofractionated whole-breast irradiation after breast-conserving surgery
Source: Clin Transl Radiat Oncol. 2025 Apr 25;53:100967. doi: 10.1016/j.ctro.2025.100967 (PMC12134538; doi:10.1016/j.ctro.2025.100967)
Supplement: Supplementary Data 1 [file mmc1.docx]

| **Supplementary Table 1. Clinical characteristics before and after IPTW of patients in SeB and SIB groups.** | | | | | | | | |
| --- | --- | --- | --- | --- | --- | --- | --- | --- |
| Characteristic | Before match, Number (%) | | | | After match (IPTW), Number (%) | | | |
|  | SeB  (N = 357) | SIB  (N = 775) | *P*-value^a^ | test SMD | SeB  (N = 361) | SIB  (N = 770.8)^†^ | *P*-value^*^ | test SMD |
| Age (y) |  |  | 0.724 | 0.027 |  |  | 0.931 | 0.006 |
| ≤40 | 102 (28.6) | 212 (27.4) |  |  | 101.0 (28.0) | 213.5 (27.7) |  |  |
| >40 | 255 (71.4) | 563 (72.6) |  |  | 260.0 (72.0) | 557.3 (72.3) |  |  |
| BMI (kg/m^2^) |  |  | 0.013 | 0.191 |  |  | 0.765 | 0.051 |
| <18.5 | 13 (3.6) | 25 (3.2) |  |  | 12.1 (3.3) | 26.0 (3.4) |  |  |
| 18.5–24.9 | 235 (65.8) | 443 (57.2) |  |  | 226.8 (62.8) | 465.5 (60.4) |  |  |
| ≥25 | 109 (30.5) | 307 (39.6) |  |  | 122.2 (33.8) | 279.3 (36.2) |  |  |
| Tumor location |  |  | 0.305 | 0.066 |  |  | 0.995 | <0.001 |
| Medial or central | 152 (42.6) | 305 (39.4) |  |  | 148.2 (41.0) | 316.6 (41.1) |  |  |
| Other quadrants | 205 (57.4) | 470 (60.6) |  |  | 212.8 (59.0) | 454.2 (58.9) |  |  |
| Surgery |  |  | 0.052 | 0.138 |  |  | 0.773 | 0.021 |
| Lumpectomy and axillary dissection | 143 (40.1) | 259 (33.4) |  |  | 129.4 (35.8) | 268.5 (34.8) |  |  |
| Lumpectomy and sentinel node biopsy | 214 (59.9) | 516 (66.6) |  |  | 231.7 (64.2) | 502.2 (65.2) |  |  |
| Histologic type |  |  | 0.002 | 0.253 |  |  | 0.718 | 0.065 |
| Ductal invasive carcinoma | 320 (89.6) | 660 (85.2) |  |  | 317.2 (87.9) | 666.5 (86.5) |  |  |
| Lobular invasive carcinoma | 5 (1.4) | 48 (6.2) |  |  | 12.4 (3.4) | 36.2 (4.7) |  |  |
| Others | 32 (9.0) | 67 (8.6) |  |  | 31.5 (8.7) | 68.1 (8.8) |  |  |
| Ductal carcinoma in situ component |  |  | <0.001 | 0.340 |  |  | 0.444 | 0.058 |
| No | 266 (74.5) | 455 (58.7) |  |  | 217.0 (60.1) | 485.1 (62.9) |  |  |
| Yes | 91 (25.5) | 320 (41.3) |  |  | 144.0 (39.9) | 285.7 (37.1) |  |  |
| Stage group (AJCC 7.0) |  |  | 0.006 | 0.212 |  |  | 0.885 | 0.038 |
| IA | 239 (66.9) | 411 (53.0) |  |  | 238.9 (66.2) | 472.5 (61.3) |  |  |
| IIA | 87 (24.4) | 237 (30.6) |  |  | 81.1 (22.5) | 181.1 (23.5) |  |  |
| IIB | 19 (5.3) | 62 (8.0) |  |  | 25.4 (7.0) | 57.8 (7.5) |  |  |
| III | 12 (3.4) | 65 (8.4) |  |  | 24.6 (6.8) | 59.4 (7.7) |  |  |
| Histologic grade |  |  | 0.945 | 0.040 |  |  | 0.951 | 0.043 |
| I | 30 (8.4) | 64 (8.3) |  |  | 32.5 (9.0) | 65.7 (8.5) |  |  |
| II | 191 (53.5) | 405 (52.3) |  |  | 183.1 (50.7) | 405.5 (52.6) |  |  |
| III | 100 (28.0) | 219 (28.3) |  |  | 102.1 (28.3) | 214.7 (27.9) |  |  |
| Unknown | 36 (10.1) | 87 (11.2) |  |  | 43.4 (12.0) | 84.9 (11.0) |  |  |
| Lymphovascular invasion |  |  | <0.001 | 0.331 |  |  | 0.940 | 0.006 |
| No | 320 (89.6) | 601 (77.5) |  |  | 292.5 (81.0) | 626.4 (81.3) |  |  |
| Yes | 37 (10.4) | 174 (22.5) |  |  | 68.6 (19.0) | 144.4 (18.7) |  |  |
| Molecular subtype |  |  | 0.115 | 0.136 |  |  | 0.952 | 0.024 |
| HR+ HER2- | 258 (72.3) | 522 (67.4) |  |  | 247.5 (68.6) | 532.6 (69.1) |  |  |
| HER2+ | 60 (16.8) | 134 (17.3) |  |  | 64.1 (17.8) | 130.2 (16.9) |  |  |
| Triple-negative | 39 (10.9) | 119 (15.4) |  |  | 49.4 (13.7) | 107.9 (14.0) |  |  |
| Chemotherapy |  |  | 0.014 | 0.159 |  |  | 0.848 | 0.013 |
| No | 111 (31.1) | 186 (24.0) |  |  | 93.4 (25.9) | 203.8 (26.4) |  |  |
| Yes | 246 (68.9) | 589 (76.0) |  |  | 267.6 (74.1) | 566.9 (73.6) |  |  |
| HER2 status and anti-HER2 targeted therapy (%) |  |  | <0.001 | 0.359 |  |  | 0.946 | 0.024 |
| Her2+ without targeted therapy | 27 (7.6) | 10 (1.3) |  |  | 11.5 (3.2) | 22.5 (2.9) |  |  |
| Her2+ with targeted therapy | 33 (9.2) | 124 (16.0) |  |  | 52.6 (14.6) | 107.7 (14.0) |  |  |
| HER2- | 297 (83.2) | 641 (82.7) |  |  | 296.9 (82.2) | 640.6 (83.1) |  |  |
| HR status and endocrine therapy (%) |  |  | 0.007 | 0.209 |  |  | 0.642 | 0.078 |
| HR+ without endocrine therapy | 9 (2.5) | 35 (4.5) |  |  | 20.7 (5.7) | 31.2 (4.0) |  |  |
| HR+ with endocrine therapy | 299 (83.8) | 585 (75.5) |  |  | 276.3 (76.5) | 599.8 (77.8) |  |  |
| HR- | 49 (13.7) | 155 (20.0) |  |  | 64.0 (17.7) | 139.8 (18.1) |  |  |
| Abbreviations: AJCC = American Joint Committee on Cancer; BMI = body mass index; HER2 = human epidermal growth factor receptor 2; HR = hormone receptor; IPTW = inverse probability of treatment weighting; SeB = sequential boost; SIB = simultaneous integrated boost; SMD = standardized mean difference.  *P-values are based on comparisons between SeB and SIB group.  ^†^The patient numbers after IPTW are weighted values rather than actual case counts, which is a characteristic of the IPTW method. | | | | | | | | |

| **Supplementary Table 2. Toxicities and survival outcomes in the literature.** | | | | | | | | |
| --- | --- | --- | --- | --- | --- | --- | --- | --- |
| Trial | Fraction (EQD2^†^) | Technology | RNI (%) | Toxicities (%) | | | Survival outcome (%) | |
|  |  |  |  | Grade 2 or higher skin toxicity | Breast induration | Fair or poor cosmesis | 5-year LRC | 5-year OS |
| Formenti 2007 ^[37]^  (n=90) | WBI 40.5Gy/15f (45.2)  SIB 48Gy/15f (57.6) | IMRT | No | 9.0 | 51.6  (90 days) | - | - | - |
|  |  |  |  |  |  |  |  |  |
| Morganti 2009 ^[17]^  (n=332) | WBI 50.4Gy/28f (48.7)  SeB 10Gy/4f (59.3) | 3D-CRT or IMRT | 16.8 | 36.7^*^ | - | - | 100.0  (31-month) | - |
|  | WBI 50Gy/25f (50.0)  SIB 60Gy/25f (60.0) |  | 28.4 | 47.1^*^ | - | - | 100.0  (31-month) | - |
|  | WBI 40Gy/16f (43.3)  SIB 44Gy/16f (49.5) |  | No | 14.1^*^ | - | - | 100.0  (31-month) | - |
|  |  |  |  |  |  |  |  |  |
| Chadha 2012 ^[18,35]^ (n=160) | WBI 40.5Gy/15f (45.2)  SIB 45Gy/15f (52.5) | 3D-CRT, IMRT, or electron | No | 24.0^*^ | - | - | 99.0 | 90.0 |
|  | WBI 46.8Gy /26f (45.2)  SeB 14Gy/7f (59.2) |  |  | 4.0^*^ | - | - | - | - |
|  |  |  |  |  |  |  |  |  |
| Raza 2012 ^[19]^  (n=169) | WBI 46Gy/23f (46.0)  SeB 14Gy/7f (60.0) | 3D-CRT or IMRT or electron | No | 72.6 | 8.2 | - | - | - |
|  | WBI 50Gy/25f (50.0)  SIB 56.25Gy /25f (58.6) |  |  | 73.0^*^ | 3.1 | - | - | - |
|  | WBI 40.5Gy/15f (45.2)  SIB 48Gy/15f (57.6) |  |  | 9.0^*^ | 24.0 | - | - | - |
|  |  |  |  |  |  |  |  |  |
| Scorsetti 2012 ^[38]^  (n=50) | WBI 40.5Gy/15f (45.2)  SIB 48Gy/15f (57.6) | VMAT | No | 2.0 | - | 0 | - | - |
|  |  |  |  |  |  |  |  |  |
| Cante 2013 ^[39,40]^  (n=872) | WBI 45Gy/20f (46.9)  SIB 50Gy/20f (54.2) | 3D-CRT | No | 32.0 | 12.8  (5-year) | 7.4  (5-year) | 100 | 97.6 |
|  |  |  |  |  |  |  |  |  |
| Dellas 2014 ^[41]^  (n=151) | WBI 40Gy/16f (43.3)  SIB 48Gy/16f (56.0) | 3D-CRT or IMRT | No | 7.8 | - | - | - | - |
|  |  |  |  |  |  |  |  |  |
| Osa 2014 ^[42]^  (n=404) | WBI 40.5Gy/15f (45.2)  SIB 48Gy/15f (57.6) | IMRT | No | - | 5.3  (5-year) | 18.0  (5-year) | 99.5 | 98.7 |
|  |  |  |  |  |  |  |  |  |
| Cooper 2016 ^[29]^  (n=400) | WBI 40.5Gy/15f (45.2)  SIB 46Gy/15f (54.2) | IMRT | No | 6.4 | - | 20.3  (4-year) | 99.0  (4-year) | - |
|  | WBI 40.5Gy/15f (45.2)  SIB 6Gy/3f (51.2) qw |  |  | 3.5 | - | 12.3  (4-year) | 98.5  (4-year) | - |
|  |  |  |  |  |  |  |  |  |
| Ghannam 2016 ^[43]^  (n=122) | WBI 45Gy/20f (46.9)  SIB 50Gy/20f (54.2) | 3D-CRT; electron for boost | No | 26.0 | - | 3.0  (1-year) | 98.4  (3-year) | - |
|  |  |  |  |  |  |  |  |  |
| Paelinck 2017 ^[21,25]^  (n=167) | WBI 40.05Gy /15f (44.5)  SeB 10-14.88Gy /4-6f (55.3-60.6) | IMRT | No | 45.8^*^ | 12.7(out of tumor bed); 9.1(tumor bed)  (2-year) | - | - | - |
|  | WBI 40.05Gy/15f (44.5)  SIB 46.8-49.95Gy/15f (55.5-61.0) |  |  | 28.9^*^ | 13.0(out of tumor bed); 7.2(tumor bed)  (2-year) | - | - | - |
|  |  |  |  |  |  |  |  |  |
| Cante 2017 ^[44]^  (n=178) | WBI 45Gy/20f (46.9)  SIB 50Gy/20f (54.2) | 3D-CRT | No | - | 7.0 | 12.2 | 95.5  (10-year) | 92.2  (10-year) |
|  |  |  |  |  |  |  |  |  |
| Fiorentino 2018 ^[20]^  (n=80) | WBI 50Gy/25f (50.0)  SIB 60Gy/25f (60.0) | IMRT or VMAT | No | 25.0^*^ | 10.0 | 7.5 | - | - |
|  | WBI 40.5Gy/15f (45.2)  SIB 48Gy/15f (57.6) |  |  | 2.5^*^ | 5.0 | 2.5 | - | - |
|  |  |  |  |  |  |  |  |  |
| Bautista Hernandez 2018 ^[45]^  (n=34) | WBI 45Gy/20f (46.9)  SIB 56Gy/20f (63.5) | IMRT | - | 0 | 0  (6-month) | 18.0  (6-month) | 100.0  (4-year) | 100.0  (4-year) |
|  |  |  |  |  |  |  |  |  |
| Lertbutsayanukul 2020 ^[30]^  (n=114) | WBI 43.2Gy/16f (48.2)  SIB 52.8Gy/16f (64.2) | 3D-CRT; electron for boost | 5.0 | - | 0 | 12.3 | - | - |
|  | WBI 50Gy/25f (50.0)  SIB 60Gy/25f (60.0) |  | 23.0 | - | 1.8 | 26.4 | - | - |
|  |  |  |  |  |  |  |  |  |
| De Rose 2020 ^[22]^  (n=831) | WBI 40.5Gy/15f (45.2)  SIB 48Gy/15f (57.6) | VMAT | - | 15.2 | 0.3  (5-year) | 1.7 | - | - |
|  |  |  |  |  |  |  |  |  |
| Saksornchai 2021 ^[36]^  (n=73) | WBI 43.2Gy/16f (48.2)  SIB 52.8Gy/16f (64.2) | 2D or 3D-CRT; electron for boost | No | - | - | - | 94.4  (10-year) | 91.6  (10-year) |
|  | WBI 50Gy/25f (50.0)  SeB 10-16Gy/5-8f (60.0-66.0) |  |  | - | - | - | 93.9  (10-year) | 91.9  (10-year) |
|  |  |  |  |  |  |  |  |  |
| Krug 2021 ^[46]^  (n=149) | WBI 40Gy/16f (43.3)  SIB 48Gy/16f (56.0) | 3D-CRT or IMRT | No | 14.7 | - | 7.0  (6-month) | - | - |
|  |  |  |  |  |  |  |  |  |
| Dicuonzo 2021 ^[47]^  (n=287) | WBI 40.5Gy/15f (45.2)  SIB 48Gy/15f (57.6) | Tomo | - | 46.0 | 16.0  (1-year) | 14.0  (1-year) | - | - |
|  |  |  |  |  |  |  |  |  |
| Vicini 2022 ^[14]^  (n=2262) | WBI 40Gy/15f (44.5)  SIB 48Gy/15f (57.6) | 3D-CRT or IMRT | No | - | - | 16.0  (3-year) | - | - |
|  | WBI 50Gy/25f (50.0) or 42.7Gy/16f (47.5)  SeB 12-14Gy/6-7f (62-64 or 59.5-61.5) |  |  | - | - | 14.0  (3-year) | - | - |
|  |  |  |  |  |  |  |  |  |
| Yadav 2022 ^[48]^  (n=27) | WBI 34Gy /10f (41.9)  SIB 40Gy /10f (53.3) | VMAT | 74.0 | 18.5 | 3.7  (4-year) | 0  (4-year) | 96.3  (4-year) | 92.0  (4-year) |
|  |  |  |  |  |  |  |  |  |
| Coles 2023 ^[13]^  (n=2617) | WBI 40Gy/15f (44.5)  SeB 16Gy/8f (60.5) | IMRT | 10.7 | - | 11.5  (5-year) | - | 97.0 | 95.9 |
|  | WBI 36Gy/15f (38.4)  Partial 40Gy/15f (44.5)  SIB 48Gy/15f (57.6) |  | 10.3 | - | 10.6  (5-year) | - | 96.9 | 95.0 |
|  | WBI 36Gy/15f (38.4)  Partial 40Gy/15f (44.5)  SIB 53Gy/15f (66.5) |  | 10.0 | - | 15.5  (5-year) | - | 95.3 | 93.3 |
|  |  |  |  |  |  |  |  |  |
| Krug 2024 ^[15]^  (n=2310) | WBI 40Gy/16f (43.3)  SIB 48Gy/16f (56.0) | - | No | - | 7.3  (5-year) | - | - | 98.2 |
|  | WBI 50.4Gy/28f (48.7)  or 42.56Gy/16f  SeB 10-16Gy/5-8f  Or  WBI 50.4Gy/28f (48.7)  SIB 58.8-63Gy/28f |  |  | - | 8.4  (5-year) | - | - | 97.9 |
|  |  |  |  |  |  |  |  |  |
| Present study  (n=1132) | WBI 43.5Gy/15f (50.0)  SIB 49.5Gy/15f (60.2) | 3D-CRT or IMRT or VMAT or electron | 15.9 | 5.0 | 14.6  (5-year) | 4.8  (5-year) | 97.7 | 97.4 |
|  | WBI 43.5Gy/15f (50.0)  SeB 52.2Gy/18f (60.0) |  | 3.1 | 3.1 | 12.3  (5-year) | 10.4  (5-year) | 97.1 | 97.1 |
| Abbreviations: 3DCRT = three-dimensional conformal radiotherapy, EQD2 = equivalent dose in 2 Gy fractions; IMRT = intensity-modulated radiotherapy; LRC = locoregional control; OS = overall survival; RNI (%) = regional nodal irradiation percentage; SeB = sequential boost; SIB = simultaneous integrated boost; VMAT = volumetric modulated arc therapy; WBI = whole-breast irradiation.  ^*^Indicates a statistically significant result for the specified outcome.  ^†^EQD2 was calculated with α/β = 4 Gy. | | | | | | | | |


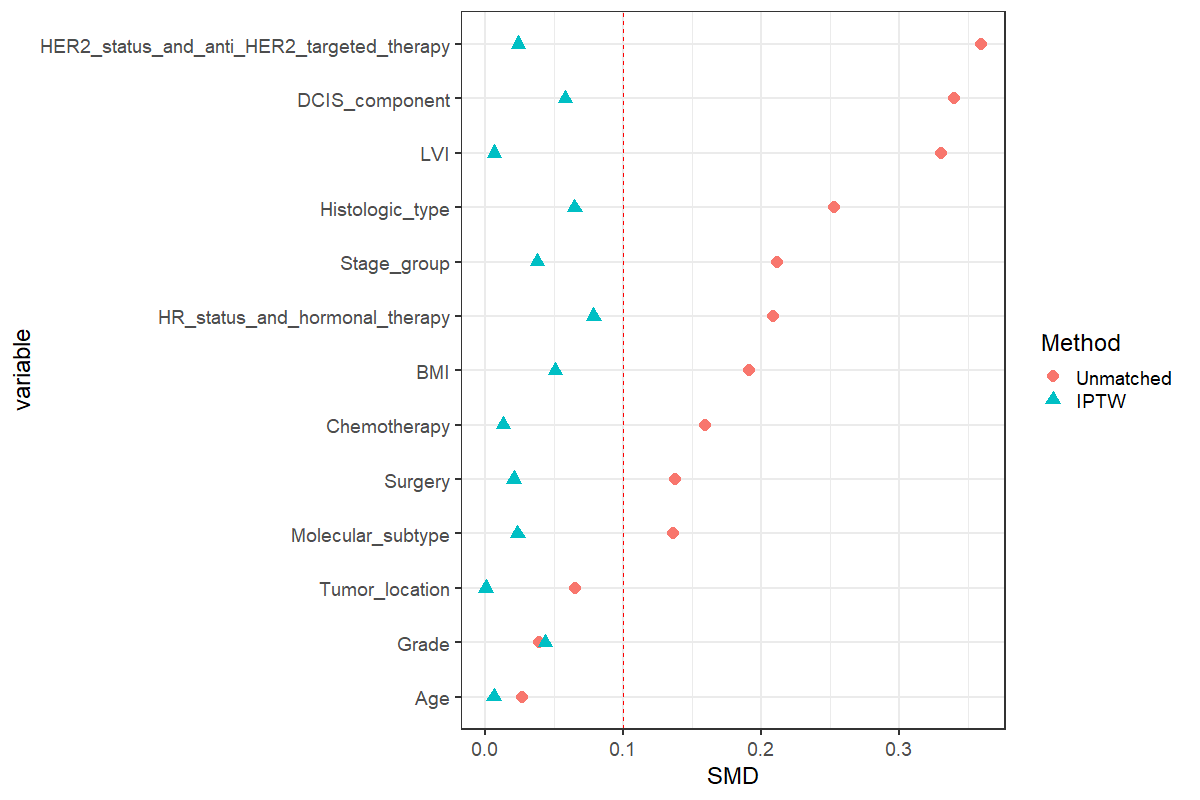


**Supplementary Figure 1. Standardized mean difference (SMD) of variables before and after IPTW. Red vertical dotted line at SMD 0.1 separates balanced (left of line) from unbalanced covariate distributions.**
